# Experience repatriation of citizens from epicentre using commercial flights during COVID-19 pandemic

**DOI:** 10.1186/s12245-020-00308-7

**Published:** 2020-10-28

**Authors:** Sarah Shaikh Abdul Karim, Fariza Anis Md Tahir, Umul Khair Mohamad, Marlina Abu Bakar, Khairul Nizam Mohamad, Maria Suleiman, Hussein Omar Khan, Julina Md Noor

**Affiliations:** 1 Prehospital Care Services Unit, Hospital Sg Buloh, 47000 Sg Buloh, Selangor Malaysia; 2 Prehospital Care Services Unit, Hospital Serdang, Kajang, Selangor Malaysia; 3 Prehospital Care Services Unit, Hospital Kuala Lumpur, Kuala Lumpur, Malaysia; 4 Emergency and Trauma Department, Hospital Kemaman, Chukai, Terengganu Malaysia; 5 Disaster, Outbreak, Crisis and Emergency Sector, Disease Control Division, Ministry of Health Malaysia, Putrajaya, Malaysia; 6 National Disaster Management Agency, Putrajaya, Malaysia; 7 Emergency Department, Faculty of Medicine, Universiti Teknologi MARA, Shah Alam, Selangor Malaysia

**Keywords:** Aircraft, Disaster medicine planning, Infectious disease medicine, Prevention and control, SARS coronavirus, Transport medicine

## Abstract

**Background:**

During the COVID-19 pandemic, many countries instituted closure of borders from international and local travels. Stranded citizens appeal to their governments to embark on citizen repatriation missions. Between February and April 2020, the Government of Malaysia directed repatriation of its citizens from China, Iran, Italy and Indonesia. We describe the preparation and execution of the repatriation mission using chartered commercial aircraft. The mission objectives were to repatriate as many citizens based on aircraft capacity and prevent onboard transmission of the disease to flight personnel.

**Results:**

Five repatriation missions performed was led by the National Agency for Disaster Management (NADMA) with the Ministry of Health providing technical expertise. A total of 432 citizens were repatriated from the missions. The operations were divided into four phases: the pre-boarding screening phase, the boarding and in-flight phase, the reception phase and the quarantine phase. The commercial aircraft used were from two different commercial airlines. Each mission had flight crew members between 10 and 17 people. There were 82 positive cases detected among the repatriated citizens. There was a single positive case of a healthcare worker involved in the mission, based on the sample taken on arrival of the flight. There were no infections involving flight team members.

**Conclusion:**

Medical flight crew must be familiar with aircraft fittings that differ from one commercial airline to another as it influences infection control practices. A clear understanding of socio-political situation of a country, transmission routes of a pathogen, disease presentation, and knowledge of aviation procedures, aircraft engineering and design is of great importance in preparing for such missions. Our approach of multidiscipline team involvement managed to allow us to provide and execute the operations successfully.

## Background

On the 30th of January 2020, the World Health Organization (WHO) declared the SARS-2 coronavirus infection as a public health emergency of international concern [1]. This was followed by declaration of Pandemic on the 11th of March 2020 [1]. Being a novel coronavirus pandemic, infectious disease and public health experts learned about the virus as the infection spreads.

On the 23rd of January 2020, the Chinese government imposed a strict lockdown to the Hubei province, to prevent the spread of infection, which was successful [2]. Air and sea travels were some factors associated with the spread of the virus from one region of the globe to another [3]. As the infection spread beyond China, many healthcare services struggled to cope with the surge in demand for in-patients and critical care interventions. Many countries adopted the Chinese government’s earlier intervention imposing lockdown on economic activities, local and international travellers, and students to contain the outbreak [4, 5]. The lockdown process creates social and psychological impacts on the societies as a whole [3, 6]. Coupled with concern about their citizens’ safety and wellbeing, the Malaysian Government began a repatriation mission called the Humanitarian Aid Disaster Relief (HADR) mission. This is the first HADR mission involving repatriation of potentially infected citizens from epicentres by the Malaysian Government.

The practice of repatriation of citizens afflicted with infectious disease from epicentres has been documented during the Ebola outbreak in Africa involving affected health care workers [7]. In the current pandemic, focus on repatriation were more on stranded citizens as illustrated by the government of Malaysia, Indonesia, and few others [3].

We aim to describe the various interventions taken to achieve the mission from flight preparation to arrival processing of repatriates.

## Methods

This is a retrospective description of process involving the repatriation of Malaysian citizens from various countries during the COVID-19 pandemic. This descriptive report is registered under the Malaysian National Research Registry (NMRR) with the research number NMRR-20-1508-55829.

Between February and April 2020, the Government of Malaysia directed repatriation of its citizens from China, Iran, Italy and Indonesia. The mission was led by the National Agency for Disaster Management (NADMA), with the Ministry of Health (MOH) as its technical advisor. The mission had one primary objective: to repatriate as many citizens based on aircraft capacity without endangering the safety of everyone on board the flight and back home in Malaysia. All passengers repatriated will be sent to a designated quarantine centre for a duration of 14 days.

**Fig. 1:**
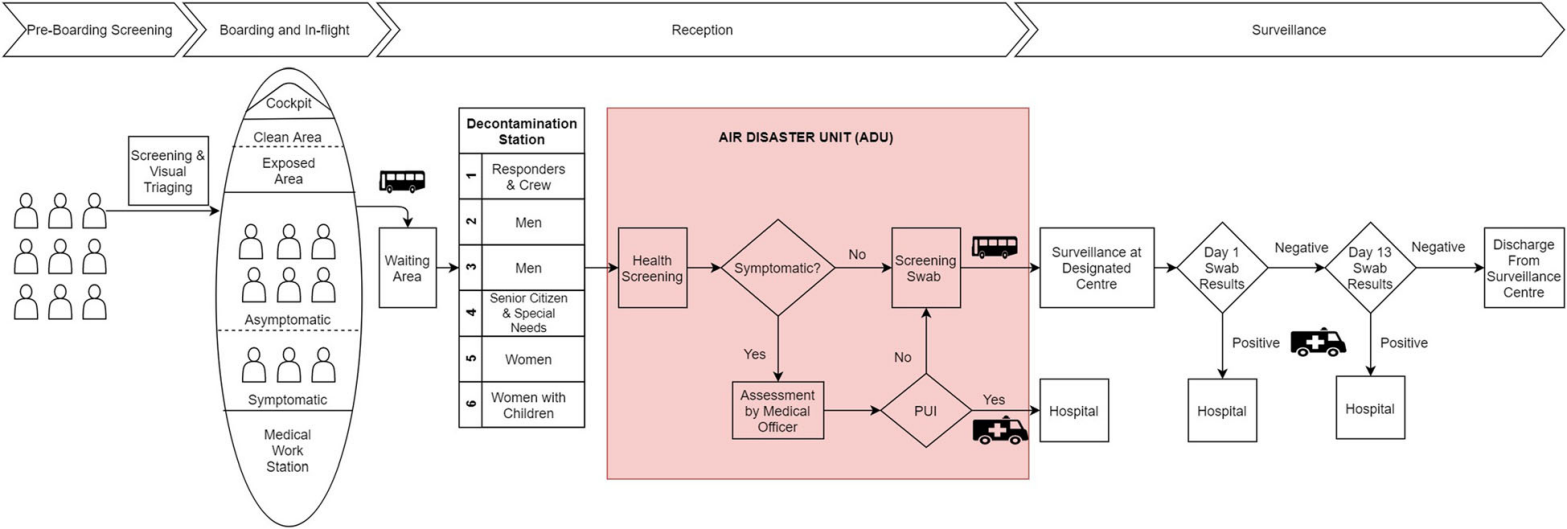
Stages of Humanitarian Assistance and Disaster Relief mission for repatriation of citizens in affected country during COVID-19

Our team is divided into three: the flight team, the reception team and the surveillance team. The operations were divided into four phases: pre-boarding screening, boarding and in-flight management, reception, and surveillance. Our team and pre-flight preparations from the first mission to Wuhan on the 3rd of February 2020 was already in line with WHO recommendations [8]. Figure 1 provides a brief description of the actions involved at each phase of response.

### Pre-flight preparation

The flight team personnel consist of a minimal airline crew required for the aircraft, an official from NADMA as the mission commander and a medical personnel from the MOH. Based on needs, other support team members including an immigration officer and officers from the Ministry of Foreign Affairs (MOFA) will be included. Medical personnel from MOH comprises of an emergency physician, a public health physician, a nurse, an assistant medical officer and an officer from the Occupational Safety and Health Unit.

All flight team personnel undergo briefing on in-flight safety procedures and use of personal protective equipment (PPE). Use of PPE differed between members of the team based on their seating location and work requirement.

Our medical team besides preparing the infection control equipment also brought along medical emergency resuscitation equipment such as a transport monitor with defibrillation capability, transport ventilator and portable ultrasound. All equipment brought were ensured to meet flight safety standards.

Citizens must register with the Malaysian Embassy of the affected country for repatriation. The Malaysian Government does not mandate the need for swab test prior to flight. Directions were given to the citizens to keep hand-carried luggage to only one light weight backpack as to reduce boarding and disembarkation time. A hygiene kit was prepared at each seat containing a Health Declaration Form, a minimum of three pieces 3-ply face masks, one hand sanitizer and a yellow biohazard-labelled bag.

### Boarding and in-flight management

All repatriates were required to wear face masks and sanitise their hands upon boarding the flight. Visual triaging was performed by medical personnel stationed at the entrance of the aircraft to identify those who appeared unwell or required special assistance. Identified repatriates were tagged and seated closer to the medical team.

**Fig. 2:**
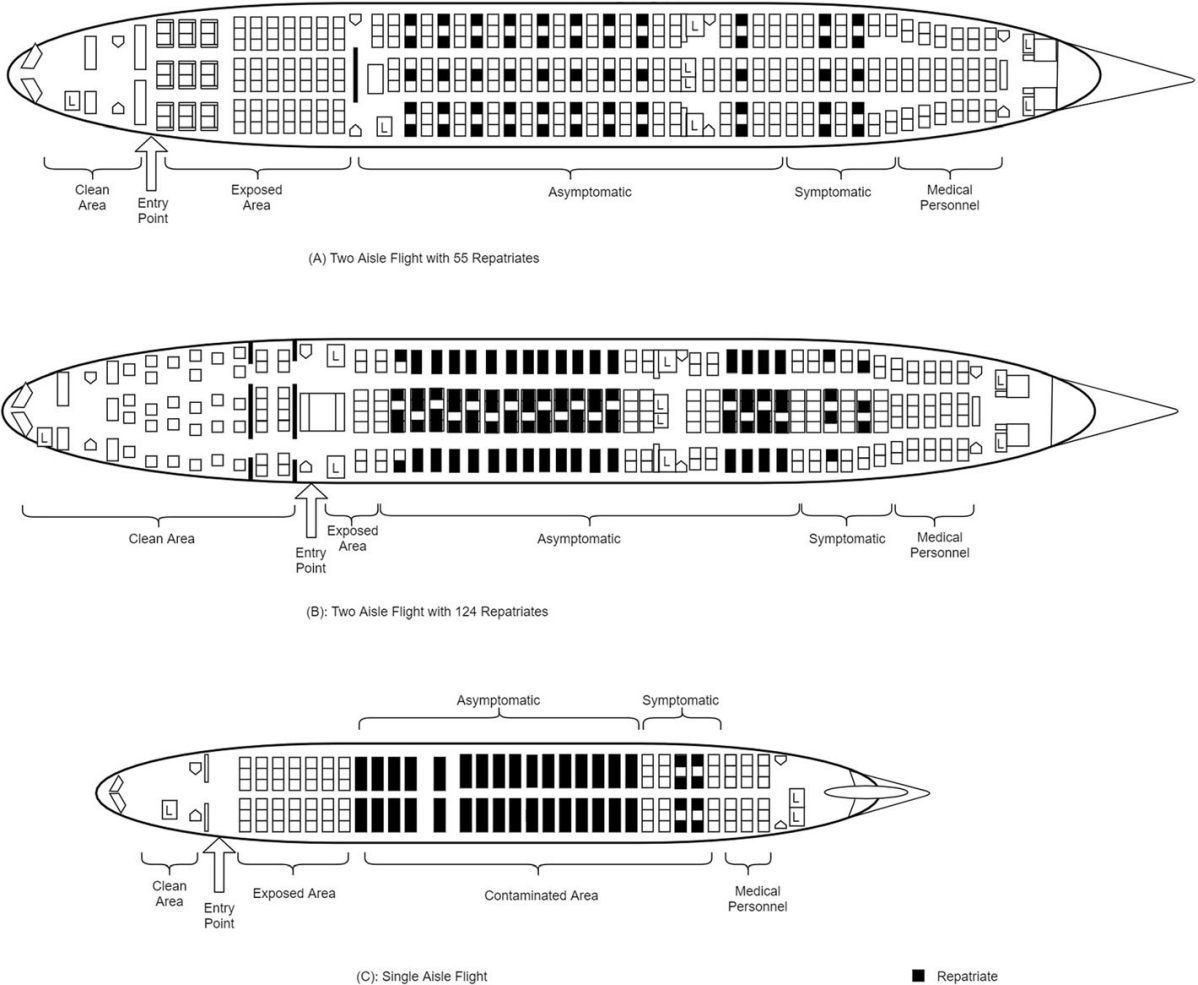
Zoning and seating arrangements of repatriates within the aircraft: (A) 2-aisle flight with 55 repatriates, (B) 2-aisle flight with 104 repatriates, and (C) 1-aisle flight

Seating arrangement considered the ability to provide distance between repatriates, separation of symptomatic and asymptomatic, and segregation from the flight crew (Fig. 2). The aircraft was divided into three zones—clean, exposed and contaminated zones. These areas were demarcated with coloured tape for ease of identification and movement restrictions. Based on feasibility and flight capacity, a two-row buffer rule of empty seats between zones was implemented to reduce risk of transmission [9]. Lavatory facilities for passengers in the clean zone and contaminated zone are separated.

The cockpit and flight crew area was kept clear from the repatriates. The exposed zone is the area where the repatriates passed through during boarding. The contaminated zone is the area where the repatriates are seated and usually extends to the rear end of the aircraft. Seating at this zone begins with asymptomatic repatriates, followed by symptomatic repatriates and lastly the medical personnel.

Our team used personal protective equipment during the flight, even though our protocol had two-row empty seats between zones. It was a practice started from the first mission and continued for all missions. There are several reports from previous PHEIC outbreaks that transmission may occur outside of the two-row rule [9–11].

Together with airline the flight crew, we prepared announcement scripts regarding the flight, implemented seating arrangements, importance of frequent hand-sanitization, use of face mask throughout the flights, minimization of movement during flight, location of dedicated lavatory facilities for passengers and importance of getting assistance for any health-related issues during the flight. All of these factors was regarded as factors that may contribute towards spread of infection during flight [12, 13].

Light meals during flight were only prepared for flight duration of more than 5 h. The type of meal was prepared by the individual airlines.

### Reception

The reception team personnel consist of officials from NADMA, personnel from the Fire and Rescue Department as Ground Mission Commander and medical personnel from the MOH. Medical personnel from MOH comprise an emergency physician, a public health physician, nurses, assistant medical officers, a pathologist and laboratory technicians. The team utilises the Air Disaster Unit (ADU) as the base of the operations. The team is tasked to perform second triaging to identify and segregate symptomatic repatriates or those requiring special assistance to MOH-assigned hospitals.

Passenger disembarkation process occurs in several groups of 40 into a bus that will ferry them from the aircraft parking bay to the ADU. Passengers seated in the clean zone were the first group to disembark, followed by the symptomatic repatriates, then asymptomatic repatriates and lastly the flight crew and medical team seated at the rear of the aircraft.

At the ADU, all repatriates undergo several processes beginning with decontamination, health screening, screening swab test and boarding of the bus that will ferry them to a designated surveillance centre. Our reception protocol followed the two-step process of identification of potential repatriates at risk of symptoms, followed by isolating for secondary evaluation and testing at designated hospital [14].

Decontamination process involved the changing of clothing from civilian attire to patient attire. Non-perishable personal items were sanitised and sealed to prevent contamination of clean areas. The change of clothing to a patient’s attire assists in the identification of repatriates from other personnel in the ADU. Six lanes were set up for decontamination to minimise crowding and waiting times. Fast track lanes for the elderly, children and responders were made available.

After the decontamination process, symptomatic repatriates or those requiring special assistance are segregated and transported to a designated hospital. Other repatriates then undergo a swab test before boarding a bus to the designated surveillance centre. Repatriates are provided with meal packs prior to boarding of the bus.

The flight team had to undergo a similar process as the repatriates except for swab test upon arrival. Swab test was only performed if any were symptomatic. However, in the mission to Indonesia, we made it compulsory for the flight team to be tested on arrival. This mission is unique and considered high risk as the repatriates are mostly religious studies students and previous border entry screening for travellers from the area had a high percentage of positive results. In all missions, the flight team had to undergo a 24-hour stay in the designated surveillance centre.

### Surveillance centre

The objective of the surveillance team is to perform daily health surveillance for presence of the infection for 14 days prior to home discharge. Repatriates will remain for a period of 14 days in the designated centres. During their stay, symptomatic repatriates tested positive on first swab were isolated and sent to a designated hospital for isolation. Besides getting access to healthcare needs, repatriates were also provided with Mental Health Psychosocial Service (MHPSS). The pandemic together with travel restrictions imposed by governments creates many uncertainty and anxiety [6].

Since the Government of Malaysia does not mandate stranded citizens in foreign country to have swab test done prior to registration for flight, there is a risk that infected repatriates may be asymptomatic upon entry screening [15]. A repeat swab was done on day 13, and results are made available prior to discharge to home.

## Results

Five repatriation missions were performed between February and the end of April 2020 involving 432 repatriates, of which 17% are children below 12 years of age. Two missions involved repatriation of Malaysians from Wuhan, China. It was then followed by a single repatriation mission from Iran, Italy and Indonesia. A summary of mission logistics is shown in Table 1.

**Table 1:** Mission team demography and logistics

Mission team demography and logistics					
Mission/HADR	1 Wuhan	2 Wuhan	3 Iran	4 Italy	5 Indonesia
Team characteristic					
Aircraft	Airbus A320-NEO	Airbus A320-NEO	Airbus A330-NEO	Airbus A330-NEO	Airbus A330-300
Flight duration	5 h	5 h	8 h	12 h	2 h 30 min
Airline crew number	12	12	14	17	10
NADMA personnel	1	1	1	1	1
Medical personnel	6	6	5	5	7
Supporting agency team	1	2	1	1	0
Total	20	21	22	24	18

All outbound flights carried only the flight team, except for the HADR mission to Italy. The mission to Italy carried 48 Italian citizens back to Italy. The aircraft underwent disinfection procedures before boarding of Malaysian citizens.

Table 2 summarises the demography and sampling results of repatriates for every mission. There were 74 positive cases detected from swab samples taken upon arrival. There were no positive results on the repeat sampling at day 13 of quarantine, except for repatriates from Indonesia, which had 8 positive results.

**Table 2:** Repatriates demography and logistics

Repatriates demography					
HADR	1 Wuhan	2 Wuhan	3 Iran	4 Italy	5 Indonesia
Total	104	68	55	81	124
Adult male	33	22	21	36	112
Adult female	46	23	22	35	7
Children < 12	25	23	12	10	5
Age grouping					
> 65	0	0	0	0	0
18 to < 65	78	45	40	71	103
12 to < 18	1	0	3	0	16
2 < 12	20	16	9	9	3
Infants	5	7	3	1	2
Transported to hospital on arrival	2	0	0	0	2
Positive on swab 1	2	0	0	0	72
Positive on swab 2	0	0	0	0	8

In the HADR mission to Indonesia, one healthcare worker (HCW) was tested positive from the first swab. All members of the flight team had to undergo a 7-day stay in the designated surveillance centre. No other flight team member was tested positive on the repeat swab. The positive HCW was discharged after 3 days of admission as subsequent testing was found to be negative.

## Discussion

When the Malaysian Government through its embassies receives a request for repatriation from Malaysians stranded in a foreign land, a needs and risk assessment is conducted. The assessment takes into consideration the country’s travel restrictions and access to flights back to Malaysia. All five missions used commercial airlines rather than military as none of the countries involved had domestic or international conflict causing safety concerns. Use of commercial airline had the advantage of ability to transport many passengers with basic comfort and existing license to operate in the affected region’s airport. However, in using commercial aircraft, there are limitations with respect to transportation of the critically ill, as the space and the fixed seats does not allow placement of a Transport Isolation System such as the one used in repatriation of highly infectious disease patients [7, 16].

The role of medical personnel in the flight team besides providing technical advice to NADMA mission commander and preparing for in-flight medical emergencies was also to ensure that infection control measures are practised throughout the flight. Our team spent time orientating the aircraft characteristics to plan standard infection control procedures during reception, boarding and in-flight. The presence of a health inspector and a public health physician allowed the mission team to advice the captain of the flight and enforce denial of boarding to any passenger deemed high risk for safety of others.

The airlines involved in the mission except that for HADR to Indonesia all had Licensed Aircraft Engineer (LAE) onboard as preparedness for any technical issues within the aircraft. One of the important aspects in prevention of infectious disease transmission within an aircraft is its ventilation system. The ventilation system in commercial aircraft is fitted with a high-efficiency particulate air (HEPA) filtration system which is effective against coronavirus [16, 17]. The filter coupled with frequent air exchanges and laminar flow pattern of air within the cabin is effective in limiting the transmission of infectious disease [13, 18]. A ventilation system failure or discontinuance of more than 30 min increases the risk of disease transmission [13, 19]. These complications were not faced by our team for all missions.

We constantly improved our planning and work processes along the way from one mission to another based on post-mission debriefings. Among the improvements that were made involved the widening the scope of our health screening tool and simplification of decontamination process.

Administration of health screening tools is widely used for travellers to assist in assessing the overall health status and risk of exposure to infectious disease and also identify those whom appear well but may have symptoms [20, 21]. In our mission, it also assists our team in identifying repatriates that require special assistance or medications during arrival and stay at the surveillance centre. Care was taken to ensure that the tool was easily understood. The HADR mission to Wuhan, China, required that the tool be translated to Simplified Chinese language. Our screening tool was not restrictive but rather inclusive to detect repatriates that require further assessment during their surveillance period [20]. An inclusive screening tool is more labour intensive for public health follow-up and monitoring which was not a problem as repatriates are all placed in a designated surveillance centre.

The Malaysian Government does not mandate swab test prior to flight. However, for HADR missions to Italy and Indonesia, the respective governments there mandated the swab test. In the HADR Indonesia mission, even though all repatriates were tested negative during pre-flight screening, the first swab performed upon arrival identified 72 positive cases. Thus, the use of such pre-test is dependent upon the type of test available, method performed and timing of the test.

The mission to Indonesia had further 8 positive cases on the second sampling process. The positive results of the second sample may occur prior to the flight as it is within the incubation period or related to the flight such as at the airport, during boarding, during disembarkation or even from fomites [12]. Our team did not investigate it as a possible secondary infection from the flight itself.

The mission to Indonesia also gave us our first positive sample result among HCW in the flight team. The sample was taken upon arrival by the reception team. The HCW was asymptomatic. A study has documented a 3% positive rate for COVID-19 among asymptomatic HCW [22]. The same study showed a cluster of positive cases among HCW within the same work area. Our Occupational Safety and Health Unit and Public Health Department conducted risk assessment and analysis for all contacts of the HCW; none were tested positive even those working in the same area. Repeated swab samplings of the HCW done at hospital were never positive. Thus, we could not deduce if the result was a false positive finding, or sampling error. We reflected on the importance of pre-mission screening of personnel for future missions especially during the peak of the pandemic.

In our two long-haul mission with flight duration of more than 5 h, consideration was made for meals to be served during the flight. Service of meal involves the opening of face mask by passengers during their meal which was a concern. Evidence for the use of mask in-flight in infectious disease control is inconclusive as compared to frequent hand hygiene [13]. Our use of light meals mitigates the need to heat up meals and prolonged periods without face masks during meals.

As the mission was considered humanitarian with the medical team as a technical advisor, there are several aviation practices which were not anticipated especially those which differ between airlines. In the HADR to Indonesia, our designated clean area was breached by a ground LAE whom had to enter the flight cockpit to do a transit check. All previous missions, an LAE was part of the flight crew negating the need of such procedure by the ground crew. Our team performed a simple disinfection of the area to allow pilots to continue their duty with minimal PPE. Thus, we would suggest future missions to also include orientation of all team members to aviation standards procedures.

## Conclusion

The WHO has provided key preparation required for repatriation of citizens during travel restrictions period due to pandemic [8]. A clear understanding of socio-political situation of a country, transmission routes of a pathogen, disease presentation and knowledge of aviation procedures, aircraft engineering and design is of great importance in preparing for such missions. Our approach of multidiscipline team involvement managed to allow us to provide and execute the operations successfully.
